# Targeting *HER2* */* *HER3* co‐mutations in metastatic breast cancer: Case reports of exceptional responders to trastuzumab and pertuzumab therapy

**DOI:** 10.1002/cnr2.1954

**Published:** 2024-03-05

**Authors:** Page E. Blas, Esther San Roman Rodriguez, Heather L. Williams, Maren K. Levin, Joshua S. K. Bell, Mariaelena Pierobon, Alexander S. Barrett, Emanuel F. Petricoin, Joyce A. O'Shaughnessy

**Affiliations:** ^1^ Clinical Oncology Research Coordination Baylor Scott and White Research Institute Dallas Texas USA; ^2^ Department of Translational Science Tempus Labs Inc. Chicago Illinois USA; ^3^ Center for Applied Proteomics and Molecular Medicine George Mason University Manassas Virginia USA; ^4^ Breast Cancer Research Program Baylor University Medical Center, Texas Oncology, US Oncology Dallas Texas USA

**Keywords:** cancer biology, exceptional responder, HER2/HER3 co‐mutations, metastatic breast cancer

## Abstract

**Background:**

Overexpression of HER2 plays an important role in cancer progression and is the target of multiple therapies in HER2‐positive breast cancer. Recent studies have also highlighted the presence of activating mutations in *HER2*, and *HER3* that are predicted to enhance HER2 downstream pathway activation in a HER2‐dependent manner.

**Methods:**

In this report, we present two exceptional responses in hormone receptor‐positive, *HER2*‐nonamplified, *HER2/HER3* co‐mutated metastatic breast cancer patients who were treated with the anti‐HER2‐directed monoclonal antibodies, trastuzumab and pertuzumab.

**Results:**

Both patients acheived exceptional responses to treatment, suggesting that combined trastuzumab, pertuzumab, and endocrine therapy could be a highly effective therapy for these patients and our observations could help prioritize trastuzumab deruxtecan as an early therapeutic choice for patients whose cancers have activating mutations in HER2.

## 1 BACKGROUND

Human epidermal growth factor receptor 2 (HER2) is overexpressed in approximately 20% of breast cancer.^1^, ^2^, ^3^ HER2‐positive breast cancers are treated successfully in both the curative and metastatic settings with the HER2‐targeted monoclonal antibodies, trastuzumab and pertuzumab.^4^, ^5^, ^6^, ^7^ Gain‐of‐function (GOF) mutations in *HER2* have been found to be present in 2%–5% of endocrine therapy‐resistant metastatic breast cancer tissues, generally in the absence of *HER2* gene amplification.^8^ Carcinogenic signaling of wild‐type HER2 in breast cancer occurs with homodimerization of HER2 in the context of HER2 amplification or via ligand dependent HER2 heterodimerization, most commonly with human epidermal growth factor 3 (HER3;^9^). While signaling of wild‐type HER2 is typically dependent on ligand interaction, mutations affecting the extracellular domain, transmembrane domain, or kinase domain of *HER2* can lead to constitutive HER2 signaling in the absence of ligand, either due to enhanced ligand‐independent kinase activity^10^ or enhanced HER2 dimerization.^11^ Breast cancers with certain *HER2* GOF mutations can be inhibited by anti‐HER2 tyrosine kinase inhibitors (TKIs),^12^ while other GOF mutations have been shown to confer resistance to TKIs.^13^ Results from the SUMMIT trial have demonstrated significant activity of the pan‐HER TKI, neratinib, alone or in combination with trastuzumab and fulvestrant, in treating non‐amplified, *HER2*‐mutated metastatic breast cancer (MBC) patients.^14^, ^15^

In contrast to HER2, wild‐type HER3 is considered to have weak kinase activity alone and carcinogenic signaling of wild‐type HER3 in breast cancer occurs mainly via ligand dependent heterodimerization with HER2, making HER3 a critical co‐receptor of HER2.^16^, ^17^, ^18^, ^19^ Mutations in the *HER3 gene* have been observed in 1%–3% of primary breast cancers.^20^ These mutations most commonly occur in the extracellular domain of *HER3*, which enhances HER2/HER3 protein dimerization even in the absence of HER3 ligand interaction, with downstream signaling being dependent on functional HER2 kinase activity.^21^, ^22^ While it has historically been unclear how patients with *HER3*‐mutant MBC will respond to HER2‐directed therapy, recent results from the SUMMIT trial demonstrated general non‐response to neratinib among patients with *HER3*‐mutant breast cancer.^14^ Conversely, a recent case study highlighted an exceptional response to trastuzumab in a patient with *HER3*‐mutated metastatic breast cancer.^22^ Other studies have identified the presence of *HER2/HER3* co‐mutations in a small subset of metastatic breast cancer patients; however, it is unknown these patients will respond to HER2‐directed therapy.^23^ Pre‐clinical studies have demonstrated that breast cancer cells with co‐occurring *HER2/HER3* mutations are inhibited by neratinib, but not by combined trastuzumab or pertuzumab.^23^ It is unknown whether these preclinical data are relevant to patients with *HER2/HER3* co‐mutant MBC and whether such patients can benefit from trastuzumab and pertuzumab therapy. Here we describe two patients with hormone receptor positive (HR+), HER2 non‐amplified, *HER2/HER3* co‐mutant MBC who have had exceptional and ongoing responses to trastuzumab and pertuzumab. We also describe the frequency of *HER2 or HER3* mutations and of *HER2/3* co‐mutations in MBC samples within the Tempus database.

## 2 METHODS

### 2.1 xT Solid Tumor Next‐Generation Sequencing Assay

The Tempus xT assay (V4) detects single nucleotide variants (SNVs), insertions/deletions (indels), and copy number variations (CNVs) in the entire coding region of 648 genes by DNA Sequencing (DNA‐Seq), in addition to targeted detection of 22 gene fusions by DNA‐Seq, and unbiased gene fusion detection by whole transcriptome RNA Sequencing (RNA‐Seq;^24^, ^25^). Somatic alterations are distinguished from incidental germline findings using a tumor‐normal match testing approach. Average depth of coverage is 500x for DNA‐Seq of tumor tissue and 150x for matched normal specimens.

### 2.2 Gene Expression Collection, Processing, and Normalization in Tempus xT Cohort

Gene expression was generated through RNA‐seq of FFPE tumor samples using an exome capture‐based protocol.^24^ Transcript‐level quantification to GRCh37 was performed using Kallisto 0.44. Transcript counts were then corrected for GC content and length using quantile normalization and adjusted for sequencing depth via a size factor method. Normalized counts in protein coding transcripts covered by the exome panel were then summed to obtain gene‐level counts. Subsequent expression analyses were performed on log10‐transformed counts.

### 2.3 Reverse Phase Protein Array (RPPA) Analysis of Laser Capture Microdissected (LCM) Tumor Epithelium

RPPA analysis was performed as previously described using lysates derived from LCM enriched tumor epithelium cell populations (~95% purity; Arcturus Pixcell IIe LCM system (Arcturus, Mountain View, CA);^23^, ^24^, ^25^, ^26^, ^27^, ^28^). Briefly, approximately 10 000 epithelial cells were captured for each sample. LCM material was stored at −80°C and cellular material lysed as described.^26^, ^27^, ^28^, ^29^, ^30^, ^31^ LCM RPPA was performed at two separate CAP/CLIA accredited RPPA laboratories, one an academic laboratory and one a commercial laboratory that in‐licensed the technology from GMU: Laboratory 1 (Center for Applied Proteomics at George Mason University) and Laboratory 2 (Theralink Technologies, Inc., Golden CO) using the exact same analytical workflow, reagents, methodology and instrumentation. RPPA analysis was performed using triplicate spot printing (approximately 10 nL per spot) onto nitrocellulose coated slides (Grace Biolabs, Bend, OR) using a Quanterix 2470 Arrayer (Quanterix, Billerica, MA). RPPA analysis was performed as previously described using florescent detection methodology under a CLIA developed and validated calibrated assay method.^32^ The RPPA data output for Laboratory 2 was generated based on individual patient values compared to a population database of similar sample specimen in order to provide both a percentile‐based score (0%–100%) and an IHC‐like ranked score (0–3+) for the sample and relative amount of each protein/phosphoprotein measured.

### 2.4 Exceptional responder case presentations

#### 2.4.1 Patient 1

A now 65‐year‐old woman was diagnosed in January 2010 at age 52 with a 1.8 cm, grade 3 invasive pleomorphic lobular carcinoma with IHC demonstrating 90% positive for both ER/PR, Ki‐67 40%, HER‐2/neu IHC equivocal (2+ in 40% of cells), and 3+ in 5% of cells. HER‐2 was negative on fluorescence in situ hybridization (FISH), with a *HER‐2/CEP17* ratio of 1.18 and average *HER2* gene copy number of 2.7. She underwent a right breast lumpectomy and was found to have an 8 mm grade 2 invasive carcinoma, with mixed lobular and ductal features, LVI absent, and a background of extensive atypical lobular hyperplasia. ER was 2–3+ positive in 95% tumor cells, PR 1–3+ positive in 80%–90% tumor cells, Ki‐67 5%–10%, HER‐2 1–2+ on IHC in 50%–75% of tumor cells. HER‐2 FISH was negative, with *HER2/CEP17* ratio of 1.68 and average *HER2* gene copy number of 4.4. The pathologist noted that approximately 23% of invasive tumor cells showed HER‐2 amplification with an average *HER2/CEP17* ratio of 3.21 and an average *HER‐2* gene copy number of 6.4. One of 8 axillary lymph nodes contained 4 mm of metastatic carcinoma. Her mother and sister had breast cancer, and her maternal grandmother may have had ovarian cancer. Germline testing revealed a *CHEK2* p.E239K variant of unknown significance.

She was treated with docetaxel and cyclophosphamide adjuvant chemotherapy followed by right breast and regional lymph node radiotherapy, as well as with adjuvant tamoxifen as she was peri‐menopausal. In March 2015, her CA 27–29 serum tumor marker was found to be elevated at 62 U/mL and abdominal CT scan showed a mass in the posterior segment of the right hepatic lobe concerning for metastatic disease. PET/CT scan showed a moderate size hypermetabolic lesion in the posterior segment of the right hepatic lobe and no other evidence of metastatic disease. Biopsy of the hepatic mass showed metastatic carcinoma, E‐cadherin positive, ER 80% positive, PR 100% positive, Ki‐67 5%, and HER‐2 negative by FISH with a *HER2/CEP17* ratio of 1.0 with an average of 3.9 HER‐2 signals per cell.

She was treated with palbociclib and letrozole from March 2015 through December 2015. Restaging PET/CT scan showed a complete metabolic response, but MRI showed a residual 1.5 cm hepatic lesion consistent with partial response. She underwent laparoscopic liver biopsy and radiofrequency ablation of the solitary hepatic lesion. Pathology revealed residual carcinoma with evidence of fibrosis. Androgen receptor (AR) IHC was evaluated in this specimen and showed 2+ expression in 60% of tumor cells.

DNA sequencing of the December 2015 metastatic liver biopsy tissue by Ashion Analytics showed gain of function mutations in *HER2* and *HER3*. The complete genomic alterations findings are summarized in Table 1. As treatment with palbociclib plus letrozole had led to a partial response, and the residual disease harbored *HER2/HER3* co‐mutations, she was treated with 6 cycles of docetaxel, carboplatin, trastuzumab and pertuzumab, followed by maintenance therapy with trastuzumab, pertuzumab and letrozole. She has remained without evidence of recurrent disease on continuous trastuzumab, pertuzumab therapy (and letrozole) since January 2016.

**TABLE 1:** Genomic mutations of patient 1 case presentation.

Lab (Assay)	Date of analysis	Sample	Patient 1	
Ashion Analytics (NGS) (% allele frequency)	**1/2016**	Liver Metastasis	ERBB2 V777L (15% allele frequency)	
			ERBB3 (E928G) (16% allele frequency)	
			Missense: BAI3, BCOR	
			Gained: BCL9, FGF3, FGF4, FGF19, GATA3, IKBKE	
			Frameshift: CDH1	
Ambry Genetics	**7/2016**	Primary BC	Germline VUS in *CHEK2* E239K	
Laboratory 1: **GMU (RPPA)**	**3/2020**	Liver Metastasis	HER2‐negative [Above cutoff: c Abl, Erk, PTEN]	
			pHER2; c Abl, Erk, HER2, p70s, PTEN	
Tempus (NGS)	**8/2020**	Liver Metastasis	Tempus xT	CDH1 c.1509_1512del p.E504fs 26.6%
				ERBB3 c.2783A > G p.E928G 22.5%
				ERBB2 c. 2329G > T p.V777L 22.3%
				Pertinent negatives: ESR1, PIK3CA, PTEN
				VUS: BCOR C.1030C > A p.H344N, ATM c.4424A > G p.Y1475C, CHEK2 c.715G > A p.E239K
Laboratory 2: Theralink® (RPPA) 2021 Assays	**8/2021**	Primary BC & Liver Metastasis	3+: HLA‐DRA (BC and liver met) 2+: p‐ERK1/2, TROP2 (liver met only) 1+: AR, p‐ERK1/2, p‐FGFR, p‐HER3, p‐JAK2, p‐mTOR, p‐PDGFRb, p‐STAT (BC), HER2, p‐PDGFRb (liver met)	

*Note*: Table 1 Primary Tumor Biopsy Collected 2/2010. Metastatic Liver Biopsy Collected 12/2015.

Abbreviations: BC, breast cancer; GMU, George Mason University; NGS, next generation sequencing; RPPA, reverse phase protein array.

**FIGURE 1:**
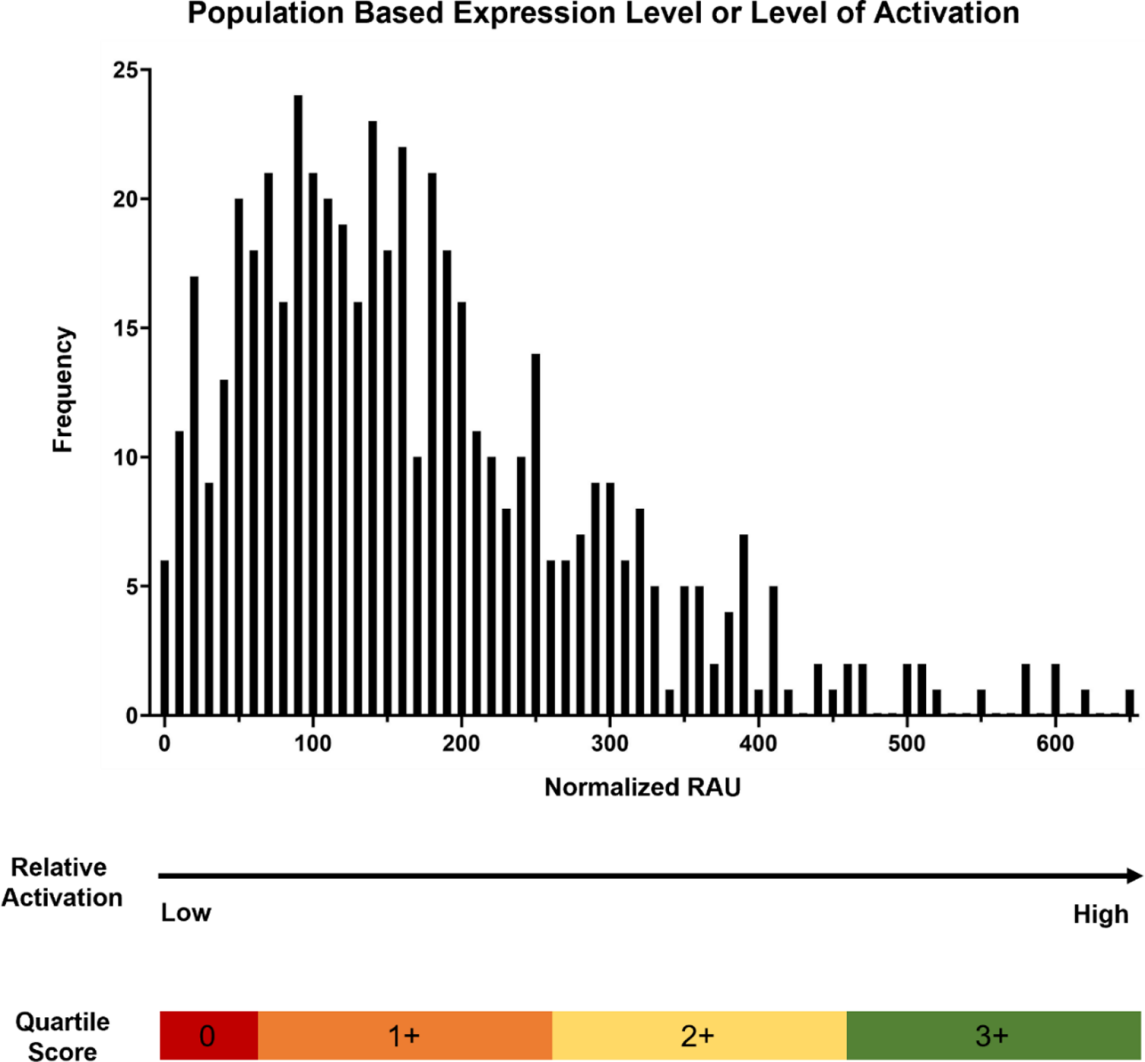
Laboratory 2: Theralink® Health Scoring Methodology. Evaluation of the activation level of protein and phosphoprotein across primary and metastatic samples was determined by extrapolating the RPPA data to a population referent obtained for each of the protein/phosphoproteins measured in order to provide both a percentile‐based score (0%–100%) of relative activation units (RAU) for each protein/phosphoprotein compared to the reference population, as well as an IHC‐like ranked score (0–3+) for the sample and protein/phosphoprotein level based on the patient‐specific value obtained by interpolation to the referent population data. An example of the population distribution of the phopshoHER2 (Y1248) levels in the referent population is shown as an example. Zero = tumors who have none (below limit of detection) to very low relative amounts of the protein/phosphoprotein (0 score = 0–10th percentile); 1+ = tumors who have low to modest relative amounts protein/phosphoprotein (11th–40th percentile); 2+ = tumors who have moderate relative amounts protein/phosphoprotein (41st–70th percentile); 3+ = tumors who have high to very high relative amounts protein/phosphoprotein (71st–100th percentile) (Figure 1).

xT DNA sequencing was subsequently performed on the 2015 metastatic liver biopsy tissue with blood normal match sequencing by Tempus and confirmed the presence of activating mutations in *HER2* and *HER3*, both with variant allelic frequency of 22%, as well as a loss of function CDH1 mutation (Table 1). The tissue was negative for homologous recombination deficiency (genome‐wide LOH was 14.5%; threshold for HRD positivity is 29%).

**FIGURE 2:**
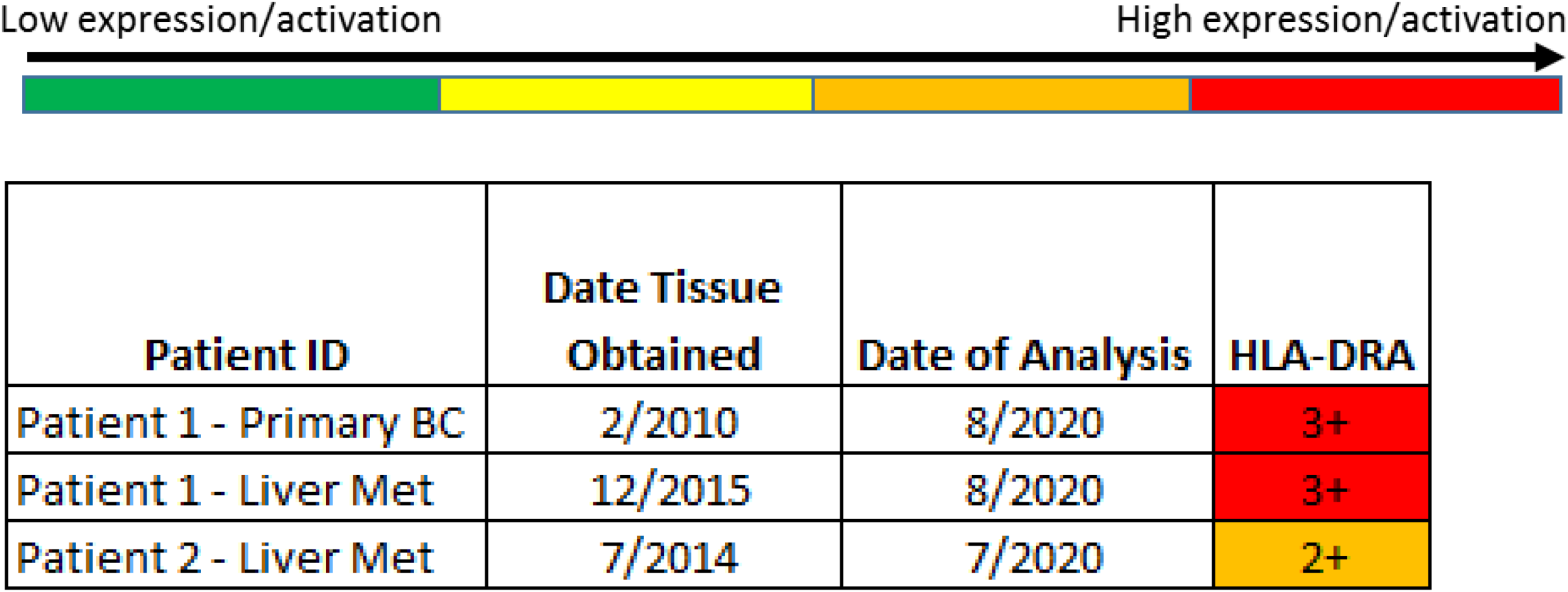
Reverse Phase Protein Array on patient tissue samples. Phosphoproteomic analysis via RPPA performed via the Theralink Assay (Laboratory 2). Strong overexpression of HLA‐DRA shown. *Source*: BC = primary breast cancer; Met = metastasis; RPPA = reverse phase protein array.

Phosphoproteomic analysis by reverse phase protein array was performed on the patient's primary breast cancer and 2015 liver metastasis at Laboratory 1 as well as at Laboratory 2 (Table 1 and Figure 2). The primary breast cancer tissue and liver metastasis showed phosphorylation and activation of the HER2 pathway, as well as activation of c‐Abl, ERK, FGFR, JAK/STAT, and the mTOR pathway. Strong overexpression of HLA‐DRA and PTEN were also noted.

#### 2.4.2 Patient 2

A now 59‐year‐old woman presented at age 36 in 1999 with a palpable left breast mass and left breast mammogram showed microcalcifications. Excisional biopsy was performed and showed 1 cm ductal carcinoma in situ, ER 70%, PR 30%, with no evidence of invasion. She had no family history of breast or ovarian cancer, and panel germline testing showed no pathogenic mutation.

She underwent bilateral mastectomy with silicone implant reconstruction. Left mastectomy showed focal residual ductal carcinoma in situ, solid and comedo types in the lower inner quadrant, with no evidence of invasion and with clear margins. In 2013, she was noted to have bilateral axillary adenopathy and breast MRI followed by biopsy revealed poorly differentiated invasive carcinoma with mixed spindle and epithelioid cells in the lower inner quadrant of the reconstructed left breast, ER‐positive and E cadherin positive. The extent of disease on MRI was 3.6 cm. Biopsy of an enlarged left breast axillary lymph node demonstrated silicone lymphadenopathy and no carcinoma. A PET/CT scan performed in September 2013 revealed multifocal metastatic disease in the liver measuring up to 10 mm in size, confirmed by MRI. Liver biopsy showed metastatic carcinoma, ER strongly positive (99%), PR weakly positive (14%), Ki‐67 47%. Pathology tested and interpreted at Neogenomics showed the biopsy to be HER2‐negative by FISH with *HER2/CEP17* ratio of 1.7 with an average of 5.6 *HER2* gene copies per cell.

She was treated with 6 cycles of docetaxel, carboplatin, trastuzumab and pertuzumab from November 2013 through February 2014 and had a partial response in the recurrent chest wall and liver disease on MRI and PET/CT scans. She then continued on therapy with trastuzumab and pertuzumab, as well as underwent oophorectomy and began letrozole. In July 2014, she underwent laparoscopic biopsy of a liver metastasis and resection of two 1 cm lesions. The remaining metastatic lesions that were not resected were 2–3 mm in size on liver MRI. Pathology revealed metastatic carcinoma, ER+ 95%, PR‐negative, Ki‐67 1%, HER2 IHC 2+ and FISH performed and interpreted at Neogenomics was equivocal with *HER2/CEP17* ratio of 2.1 with an average of 6.98 *HER2* gene copies per cell. She has continued on trastuzumab and pertuzumab (and letrozole) since 2013 with no evidence of progression of disease in liver nor chest wall on breast and abdominal MRI scans and on PET/CT scan.

**FIGURE 3:**
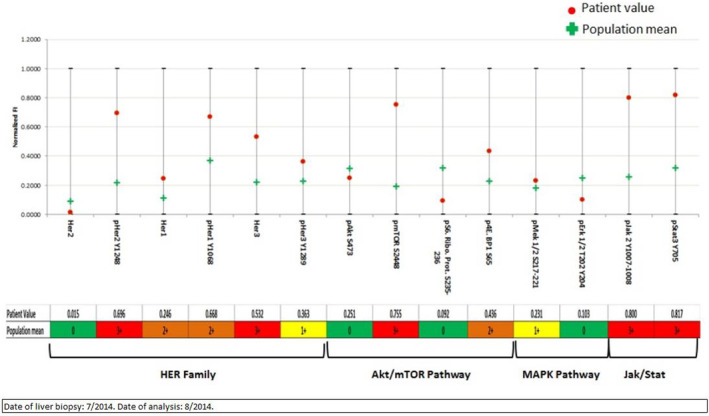
Reverse Phase Protein Array on patient 2 liver metastasis. Phosphoproteomic analysis via RPPA performed via the Theralink Assay (Laboratory 2). Activation of HER1, HER2, and HER3, strong activation of the JAK/STAT and mTOR pathways, and accumulation of 4EBP1 demonstrated.

Next generation sequencing of the July 2014 liver biopsy was performed at Molecular Health and showed somatic variants of uncertain significance in AR, HER2 and HER3 and no HER2 amplification (Table 2). Phosphoproteomic analysis via RPPA was performed on the patient's liver metastasis by Laboratory 1 in 2014 (liver biopsy of July 2014) (Figure 2) and again in August 2021 (liver biopsy from July 2014) (Figure 3). RPPA showed activation of HER1, HER2, and HER3, with strong activation of the JAK/STAT and mTOR pathways, with accumulation of 4EBP1 (Figure 3). Activation of ERK1/2, FGFR, and H2AX were also noted, as was 2+ overexpression of HLA‐DRA (Figure 2).

**TABLE 2:** Genomic mutations of Patient 2 case presentation.

Lab (Assay)	Date of analysis	Sample	Patient 2	
**Treatment MAP/Molecular Health (NGS)**	**8/2014**	Liver Metastasis	Variants of uncertain significance AR.pR619W, ERBB2.pA1216D, ERBB2.pD1058G, ERBB3.pK118R	
			JAK2.pL393V, KDR.pR57T, PDGFRA.pQ551K	
			FGFR3, FGFR4, CDK6, SHH, MYC, NOTCH1, HRA5, CCND1	
**Laboratory 2: Theralink® (RPPA)** 2014 Assay	**8/2014**	Liver Metastasis	3+: p‐HER2, p‐mTOR, HER3, p‐JAK2, p‐STAT3 2+: HER1, p‐HER1, p‐4EBP1 1+: p‐HER3, p‐MEK1/2	
**Tempus (NGS)**	**7/2020**	Liver Metastasis	Tempus xT	Amplification of ERBB2, FOXA1, GNAS, MYC, ZNF217 ERBB2 gene copies: 7
				VUS: AR, FANCA, FLT4, OLIG2, SOX10, STK11
				Pertinent negatives: ESR1, PIK3CA, PTEN
				No pathogenic germline variants found
**Laboratory 2: Theralink® (RPPA)** **2021 Assay**	**8/2021**	Liver Metastasis	2+: HLA‐DRA 1+: p‐H2AX TROP2	

*Note*: Table 2 Metastatic Liver Biopsy Collected 7/2014. Patient's primary tumor biopsy was unavailable for analysis.

Abbreviations: BC, breast cancer; NGS, next generation sequencing; RPPA, reverse phase protein array.

xT DNA sequencing was performed by Tempus on the July 2014 liver biopsy, with sequencing of blood normal matching and showed copy number gain/amplification in ERBB2 (7 copies; research use only), MYC, FOXA1, GNAS, ZNF217, and no activating mutations in *HER2* or *HER3*. Homologous recombination deficiency was not found with genome wide LOH of 22.7%; threshold for HRD positivity is 29%). Liver biopsy performed in April 2015 was negative for carcinoma.

#### 2.4.3 Analysis of *ERBB2/ERBB3* mutations in the Tempus database

**FIGURE 4:**
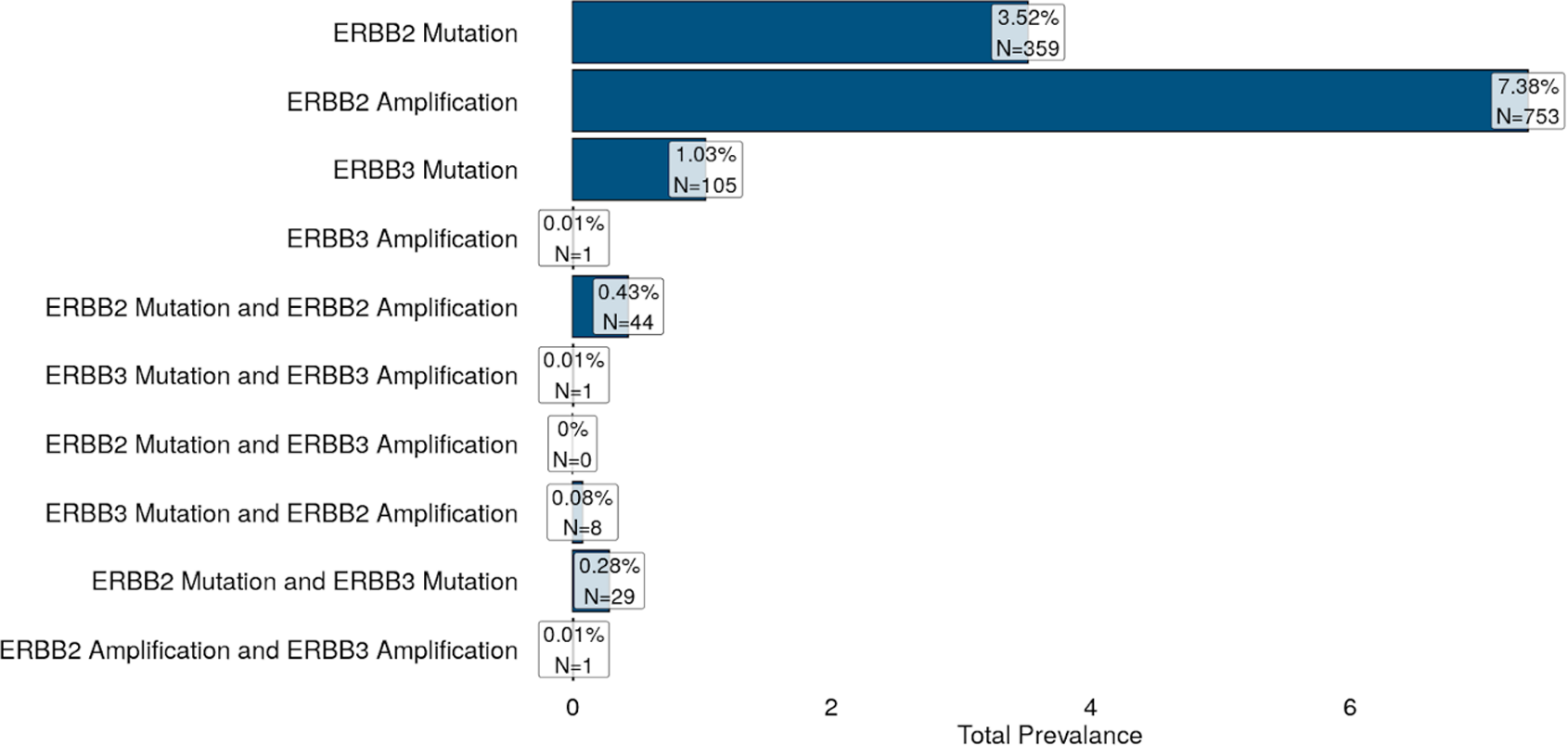
Prevalence of *HER2/HER3* alterations from the Tempus database. Analysis of 10 208 breast cancers with clinical Tempus xT reports for comparison across *ERBB2* and *ERBB3* amplification and mutatioan.

Among 10 208 breast cancers with clinical Tempus xT DNA and RNA sequencing reports, pathogenic *ERBB2* mutations (3.52%) are roughly half as common as ERBB2 amplifications (7.38%). *ERBB3* mutations occur in 1.03% of breast cancers. Intriguingly, *ERBB2* mutations are more likely to co‐occur with *ERBB2* amplification (OR = 1.8, *p* = .0008969), suggesting that amplification enhances the pathogenicity of mutations. *ERBB3* mutations are much more likely to co‐occur with *ERBB2* mutations (OR = 11.3, *p* = 1.29e‐18). Notably, the only patient with a reported *ERBB3* amplification also harbored an *ERBB2* amplification, and *ERBB3* mutations are also likely to co‐occur with *ERBB2* amplification (OR = 1.8, *p* = .0005422). These data suggest that *ERBB3* activation is more likely to be observed in the context of *ERBB2* activation and may enhance *ERBB2*‐driven oncogenesis (Figure 4).

## 3 DISCUSSION

**FIGURE 5:**
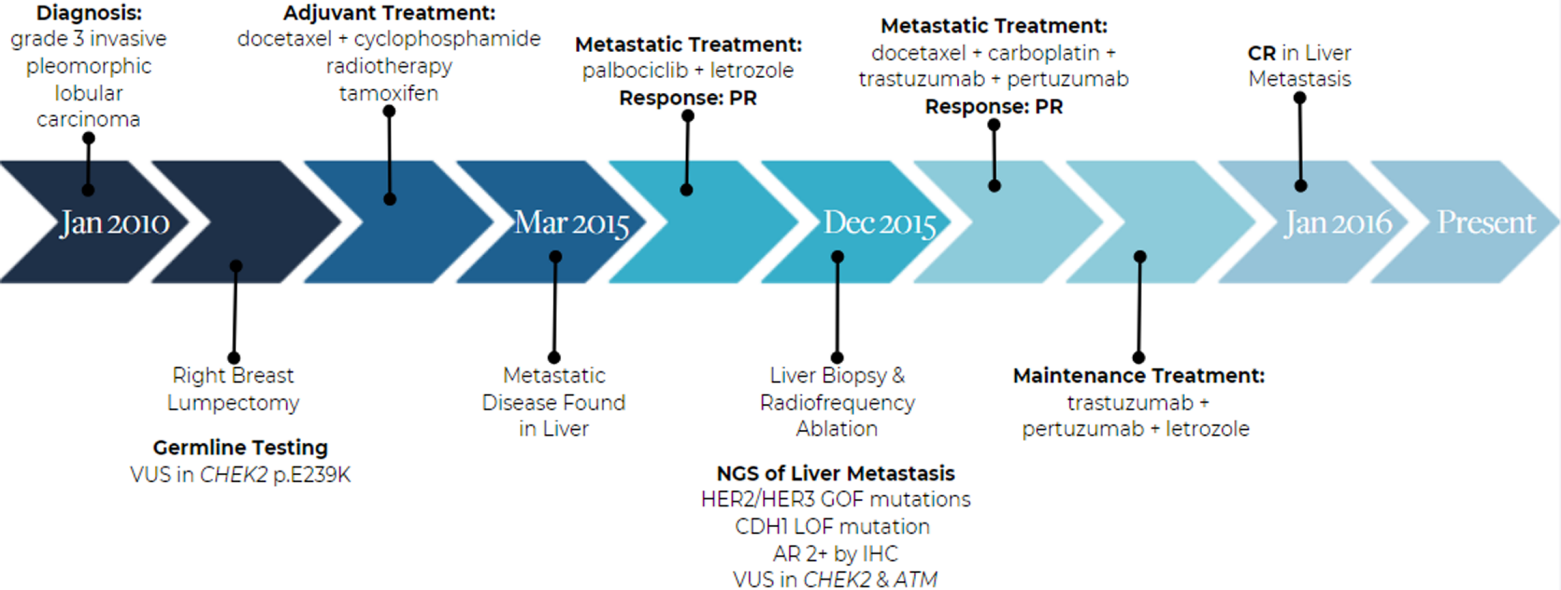
Patient 1 timeline. Treatment timeline for Patient 1 including diagnosis, procedures, treatments, best response, and key genetic findings.

**FIGURE 6:**
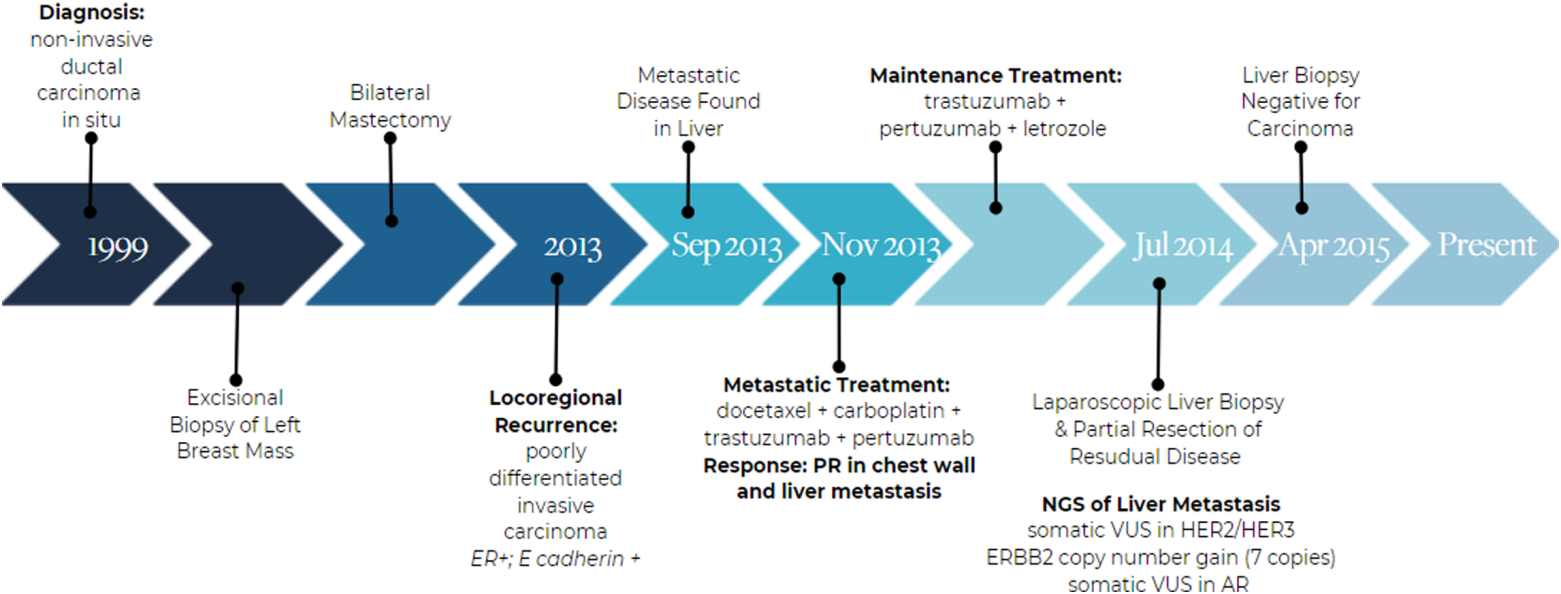
Patient 2 timeline. Treatment timeline for Patient 2 including diagnosis, procedures, treatments, best response, and key genetic findings.

The two exceptional responders described in this report have both had prolonged and ongoing benefits from trastuzumab and pertuzumab therapy, in combination with an aromatase inhibitor. Complete timelines for each patient including diagnosis, treatment, and biomarker analysis information can be found in Figures 5 and 6. Both patients were peri‐menopausal, healthy, Caucasian women with over 30 years of hormonal contraceptive use. Next generation sequencing (NGS) analysis demonstrated activating mutations in HER2 and HER3 in the setting of HER2‐ disease by conventional IHC and FISH in both patients' cancer as well as variants of unknown significance in DNA damage repair genes, *CHEK2* and *ATM*, and *FANCA* respectively. Proteomic evaluation shows that both patients' cancers had strong HLA‐DRA expression, indicating increased immunogenicity of their cancer. Additionally, reverse phase protein array of the patients' liver biopsies showed that both patients had strong activation of both p‐HER2 and p‐HER3.

Increasing utilization of tumor NGS and plasma ctDNA analyses in clinical practice are identifying an increasing number of patients with HR+, HER2 not‐amplified metastatic breast cancer that harbors co‐mutations in HER2 and HER3. The first patient's HER2 and HER3 co‐mutation status was confirmed by Tempus NGS analysis of a metastatic liver biopsy sample. NGS of patient 2's metastatic liver disease initially conducted in 2014 show somatic variants in HER2 and HER3, without HER2 amplification. Phosphoproteomic analysis supported the observation of activation of the HER family with elevated levels of phosphorylated HER1, 2, 3 with downstream pathway activation. However, NGS by Tempus in 2021 of a 2014 liver biopsy did not show activating mutations in HER2 or HER3 and showed low level HER2 gene copy number gain. It is possible that the discordance in the NGS results performed in 2014 and 2021 may have occurred because of aging of the paraffin‐ embedded tissue with deterioration in DNA quality, however, RPPA analyses done in 2014 and in 2021 also yielded differing results (Table 2) suggesting tumor heterogeneity within the biopsy/resection specimens. Neither of these patients' metastatic cancers harbored mutations in ESR1, PIK3CA or PTEN, which have been associated with resistance to HER2‐targeted therapy.^13^, ^33^ Interestingly, both patients' liver metastases showed strong overexpression HLA‐DRA on RPPA which has been shown to predict for benefit from immune checkpoint inhibitor therapy to a greater extent than does PDL‐1 expression in TNBC.^34^ This intriguing finding suggests that patients who are exceptional responders to combined trastuzumab and pertuzumab therapy may have immunologically active metastatic disease that overexpresses HLA‐DRA.

Co‐occurring activating mutations in HER2 and HER3 are known to enhance ligand independent activation of the HER2/HER3 signaling pathway independent of downstream activation by phosphoinositide 3‐kinase (PI3K) mutations.^13^ A recent analysis of the cBioPortal database showed that in breast cancers with HER2 mutations, co‐occurring mutations occurred in HER3 8.7%, HER4 10.09%, PIK3CA 18.23%, and EGFR 8.5%.^13^ Pre‐clinical studies have shown that co‐activating mutations in HER2 and HER3 are generally nonresponsive to the HER2‐targeting antibodies, trastuzumab and pertuzumab. Interestingly, patients with HR+, HER2‐non‐amplified metastatic breast cancer that contains an activating HER2 mutation are known to respond to neratinib, but prolonged benefit from neratinib, with responses lasting 24 weeks or longer, was found not to occur in patients with co‐activating mutations in HER2 and HER3.^35^ Patients with clinical benefit from neratinib with responses lasting 24 weeks longer were found to have a HER2 activating mutation in the absence of a HER3 activating mutation.^35^ In addition, patients with HR+, HER2‐ metastatic breast cancer that contained a HER3 activating mutation with wild type HER2 did not garner significant benefit from neratinib.^36^ Therefore, the accumulated evidence suggests that activating co‐mutations in HER2 and HER3 are less responsive to inhibition by HER2‐directed antibodies in preclinical models and by neratinib in the SUMMIT trial.^14^, ^15^, ^23^ Instead, a dual blockade approach for patients with HR+/HER2 mutant breast cancer may be more appropriate.^37^, ^38^

**FIGURE 7:**
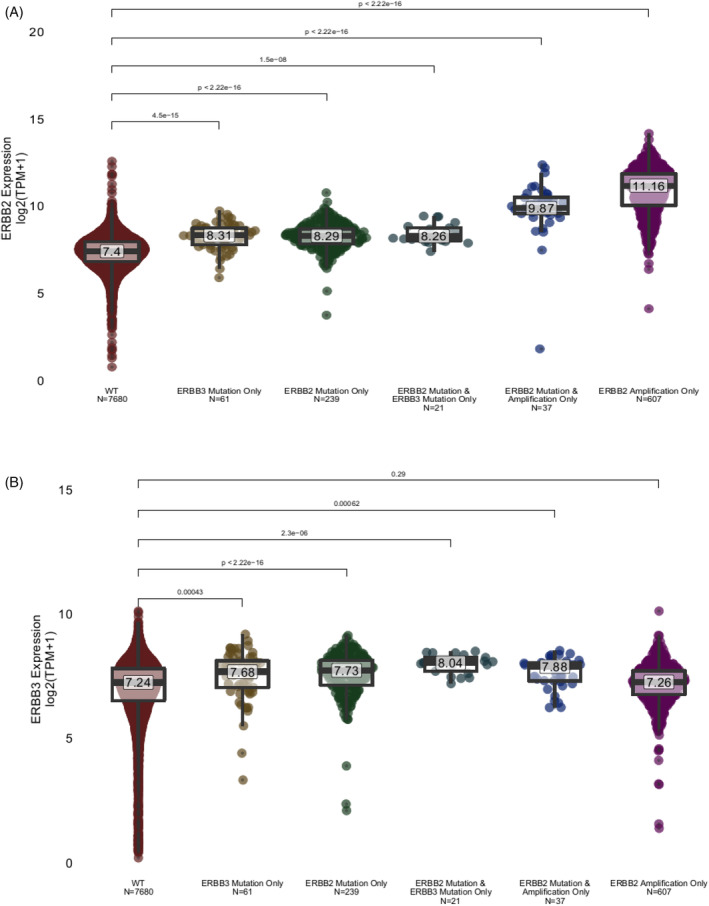
HER2 and HER3 gene expression on RNA sequencing. (A) HER2 gene expression stratified by wild type (WT), ERBB2 mutation only, ERBB3 mutation only, ERBB2/3 co‐mutation, ERBB2 mutation and amplification, and ERBB2 amplification only. (B) HER3 gene expression stratified by wild type (WT), ERBB2 mutation only, ERBB3 mutation only, ERBB2*/3* co‐mutation, *ERBB2* mutation and amplification, and *ERBB2* amplification only.

Breast cancers harboring ERBB2 mutations, ERBB3 mutations, or both, absent reportable HER2 amplification, have significantly higher HER2 expression. Tumors with ERBB2 amplification have even higher HER2 expression than mutant samples, but tumors harboring both HER2 mutation(s) and amplification had HER2 levels intermediate between mutation‐only and amplified‐only samples. This suggests that in the presence of HER2 mutations, breast cancers can be driven by lower HER2 amplification levels than seen in HER2 non‐mutant, HER2‐amplified tumors, and that higher HER2 expression may be selected for in tumors with HER3 mutations. Note that not all patients in the Tempus xT database who had ERBB2 and/or ERBB3 mutations had gene expression data available from RNA sequencing (Figure 7A).

Tumors with mutations in either ERBB2 or ERBB3, but particularly with both, displayed increased HER3 expression. Notably, samples with HER2 amplification did not display increased ERBB3 expression, and samples with both HER2 mutation and HER2 amplification display intermediate ERBB3 expression. This suggests that HER2 amplification may be an independent driver of disease, while mutations in ERBB2/ERBB3 may co‐operate and generate oncogenic clonal expansion and cause higher expression of both genes (Figure 7B). The Tempus database shows that only 0.28% of patients were found to have ERBB2 and ERBB3 co‐mutations (Figure 4). With only two exceptional responder cases presented, it is difficult to distinguish whether the main genomic alterations that underlie the mechanism of the patients' exceptional responses were due to co‐mutations in HER2 and HER3, or mutation(s) in HER2 combined with HLA‐DRA overexpression. It is possible that the upregulated expression of HLA‐DRA may have played a role in the patients' exceptional responses, as these cancers may have been more immunogenic.

**FIGURE 8:**
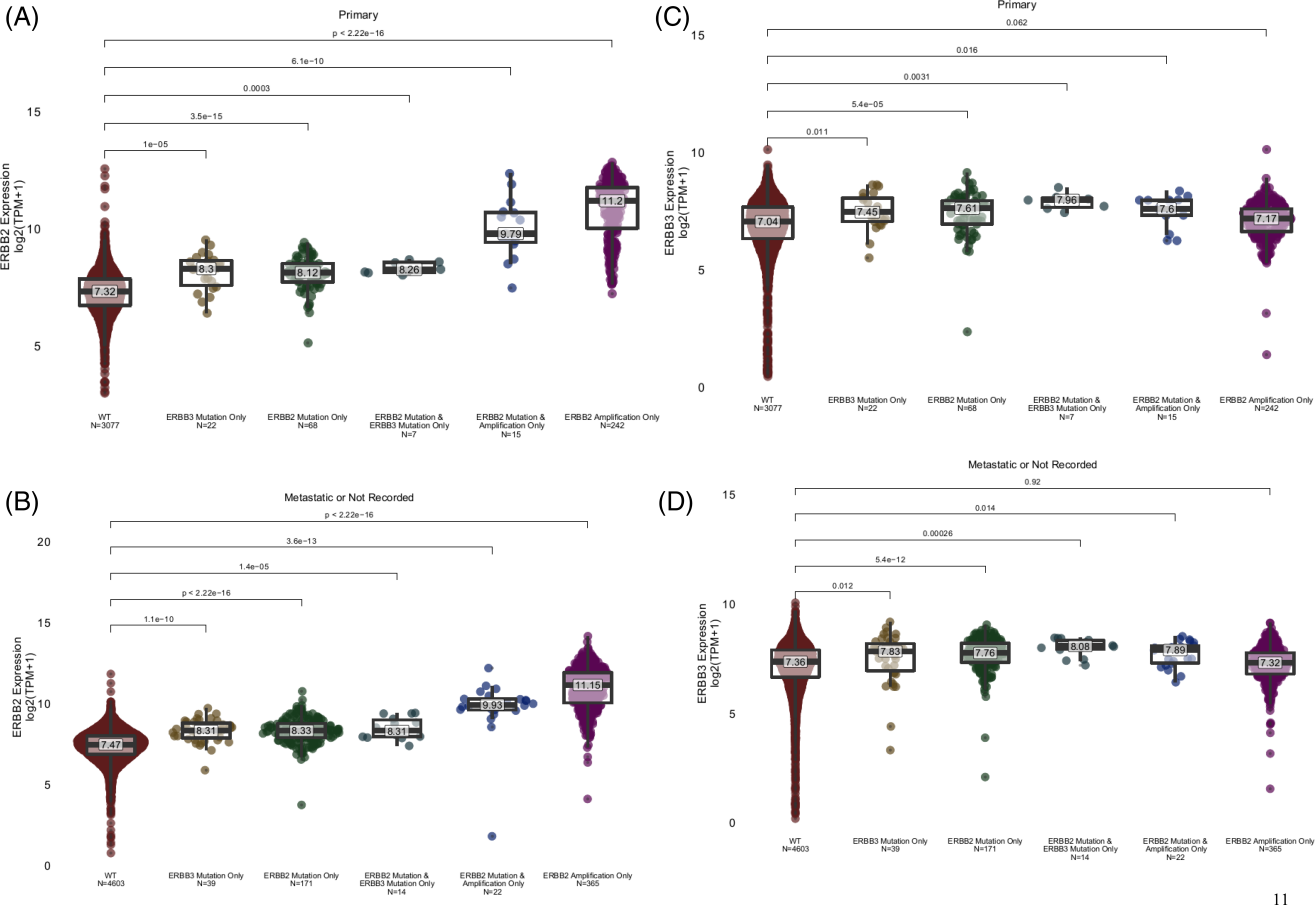
HER2 and HER3 gene expression on RNA sequencing by tumor type. (A) HER2 gene expression in the primary tumor stratified by wild type (WT), ERBB2 mutation only, ERBB3 mutation only, ERBB2/3 co‐mutation, ERBB2 mutation and amplification, and ERBB2 amplification only. (B) HER2 gene expression in the metastatic tumor stratified by wild type (WT), ERBB2 mutation only, ERBB3 mutation only, ERBB2/3 co‐mutation, ERBB2 mutation and amplification, and ERBB2 amplification only. (C) HER3 gene expression in the primary tumor stratified by wild type (WT), ERBB2 mutation only, ERBB3 mutation only, ERBB2/3 co‐mutation, ERBB2 mutation and amplification, and ERBB2 amplification only. (D) HER3 gene expression in the metastatic tumor stratified by wild type (WT), ERBB2 mutation only, ERBB3 mutation only, ERBB2/3 co‐mutation, ERBB2 mutation and amplification, and ERBB2 amplification only.

Most of the Tempus samples analyzed were obtained from metastatic breast cancer sites. Cancer gene expression analyses in metastases can be confounded if normal tissue in the metastatic organ site over‐expresses genes of interest. However, the ERBB2 and ERBB3 expression changes we observed when the genes had activating mutations or were amplified are seen in both primary and metastatic biopsy tissues, corroborating the findings in the overall cohort (Figure 8A–D).

Our analysis of HER2 expression levels in HER2 and/or HER3 mutant metastatic breast cancer is of interest in the context of the clinical availability of trastuzumab deruxtecan as treatment for patients with HER2 low metastatic breast cancer, and for metastatic lung cancer patients whose cancers harbor an activating HER2 mutation. Our analyses showed that breast cancers that harbor activating mutations in both HER2 and HER3 have significantly higher HER2 expression levels compared with breast cancers that do not contain these mutations. As shown in Figure 7A, breast cancers, primary or metastatic, that harbor a HER2 activating mutation, with or without an activating mutation in HER3, demonstrate significantly higher HER2 expression levels than cancers that have wild type HER2 genes. These observations could help prioritize trastuzumab deruxtecan as an early therapeutic choice for patients whose cancers have activating mutations in HER2. There are also antibody drug conjugates under development that target HER3, and recent findings from Krop et al. from ASCO 2022 demonstrated significant activity of patritumab, an anti‐HER3 directed antibody‐drug conjugate with a deruxtecan payload in patients with metastatic breast cancers that overexpressed HER3.^39^ We found that tumors with mutations in either HER2 or HER3, but particularly with both, displayed increased HER3 expression, while breast cancers with HER2 amplification alone do not overexpress HER3.^40^

Given that activating mutations in HER2, with or without HER3 co‐mutation generally lead to overexpression of HER2, it is likely that trastuzumab deruxtecan will be the treatment choice for patients whose cancers are found to harbor HER2 activating mutations with or without HER3 co‐mutation, although clinical efficacy data demonstrating this are not yet available. A recent update of the SUMMIT trial has shown a 49% overall response rate in patients with HR+, HER2‐, CDK 4/6 inhibitor‐pretreated metastatic breast cancer with activating HER2 mutations treated with neratinib, trastuzumab, and fulvestrant.^15^ Our current report of two exceptional responders with HR+ HER2 and HER3 co‐mutant metastatic liver disease suggests that combined trastuzumab, pertuzumab and endocrine therapy can also be a highly effective therapy for these patients and is worthy of further study.

## AUTHOR CONTRIBUTIONS

**Page E. Blas:** Project administration (supporting); resources (equal); writing – review and editing (equal). **Esther San Roman Rodriguez:** Investigation (equal); resources (equal); writing – review and editing (equal). **Heather L. Williams:** Project administration (supporting); resources (equal); writing – review and editing (supporting). **Maren K. Levin:** Funding acquisition (equal); project administration (lead); resources (equal); writing – review and editing (supporting). **Joshua S. K. Bell:** Conceptualization (equal); methodology (equal); visualization (equal); writing – original draft (equal); writing – review and editing (equal). **Mariaelena Pierobon:** Data curation (equal); formal analysis (equal); validation (equal); visualization (equal); writing – original draft (equal). **Alexander Barrett:** Conceptualization (equal); writing – original draft (equal); writing – review and editing (equal). **Emanuel F. Petricoin:** Data curation (equal); formal analysis (equal); validation (equal); visualization (equal); writing – review and editing (equal). **Joyce A. O'Shaughnessy:** Conceptualization (equal); funding acquisition (equal); investigation (equal); methodology (equal); project administration (equal); supervision (equal); writing – original draft (equal); writing – review and editing (equal).

## CONFLICT OF INTEREST STATEMENT

Joshua SK Bell and Alexander Barrett are employees of and shareholders of Tempus. Joyce O'Shaughnessy has received honoraria for consulting and/or advisory boards for AbbVie Inc., Agendia, Amgen Biotechnology, Aptitude Health, AstraZeneca, Bayer, Bristol‐Myers Squibb, Carrick Therapeutics, Celgene Corporation, Clovis Oncology, Daiichi Sankyo, Eisai, G1 Therapeutics, Genentech, Gilead Sciences, GRAIL, Halozyme Therapeutics, Heron Therapeutics, Immunomedics, Ipsen Biopharmaceuticals, Lilly, Merck, Myriad, Nektar Therapeutics, Novartis, Ontada, Pfizer, Pharmacyclics, Pierre Fabre Pharmaceuticals, Puma Biotechnology, Prime Oncology, Roche, Samsung Bioepis, Sanofi, Seagen, Syndax Pharmaceuticals, Taiho Oncology, Takeda, TheraLink and Synthon.

## ETHICS STATEMENT

Written informed consent was obtained from the two exceptional responders for whom genetic information is discussed and ethical approval was obtained from the local Institutional Review Board for this study. For Tempus data, Tempus database analyses on retrospective de‐identified data was exempt from Institutional Review Board oversight and informed consent (Pro0002950).

## Data Availability

The data supporting the findings of this study are available from the corresponding author upon reasonable request.
